# Reductions in Muscle Strength and Range of Motion Cause Locomotion Disability via Locomotion-Related Functional Limitation in Japanese Older Adults: A Cross-Sectional Study

**DOI:** 10.1155/2021/6627767

**Published:** 2021-07-07

**Authors:** Hungu Jung, Shigeharu Tanaka, Yuji Iwamoto, Takashi Kawano, Masahiro Yamasaki, Ryo Tanaka

**Affiliations:** ^1^Graduate School of Integrated Arts and Sciences, Hiroshima University, 1-7-1 Kagamiyama, Higashi-Hiroshima, Hiroshima 739-8521, Japan; ^2^Department of Sports, Health and Well-being, Faculty of Human Health Science, Hiroshima Bunka Gakuen University, 3-3-20 Heiseigahama, Akigunsakacho, Hiroshima 731-4312, Japan; ^3^Physical Therapy Major, School of Rehabilitation, Kanagawa University of Human Services, Yokosuka 238-8522, Japan

## Abstract

**Background:**

Functional issues (impairments, functional limitations, and disabilities) gradually occur with age. Nonetheless, maintaining physical capability may help prevent locomotion disabilities at an older age. The present study aimed to determine whether reductions in muscle strength and range of motion (ROM) cause locomotion disability via locomotion-related functional limitations among healthy older adults.

**Methods:**

Data from a total of 144 participants (61 men, 83 women) were analyzed. To assess locomotion disability, the locomotor domain of the activities of daily living (ADLs) survey from the Ministry of Education, Culture, Sports, Science, and Technology of Japan was used. Muscle strength (grip strength) and two ROMs (hip flexion and knee flexion) were measured. To measure locomotion-related functional limitations, participants underwent a 10 m hurdle walking test and side-step test. Thereafter, path analysis was conducted for testing the hypothetical model. The goodness of fit in the model was assessed using statistical parameters, such as the chi-square value, goodness of fit index (GFI), adjusted goodness of fit index (AGFI), comparative fit index (CFI), and root mean square error of approximation (RMSEA).

**Results:**

The analysis revealed a nonsignificant chi-square value (chi-square = 41.885; *p*=0.113), as well as high values of GFI (0.944), AGFI (0.904), CFI (0.970), and RMSEA (0.046), indicating that locomotion disability was caused by locomotion-related functional limitations, which were influenced by muscle strength and ROM.

**Conclusions:**

The present study demonstrated that decreased muscle strength and ROM caused locomotion disability via locomotion-related functional limitations. Older adults should participate in physical exercise programs that focus on strengthening muscles and improving ROM to counteract age-related locomotion disability.

## 1. Introduction

Activities of daily living (ADLs) refer to an individual's ability to perform activities including eating, using the toilet, transferring, dressing, and bathing that are necessary for survival [1]. A self-reported ADL survey detected early “difficulty” or “dependency” during ADL tasks, such as locomotion, among older adults living in the community [2]. Locomotion refers to the individual's ability to effectively move within their environment and includes physical activities, such as walking, turning, and stair climbing [3]. Considering that locomotion disability has been associated with restrictions in leisure activities and community participation [4, 5], maintaining good locomotive function is imperative, particularly among healthy older individuals without ADL disabilities.

Physical capabilities, such as muscle strength and range of motion (ROM), markedly decrease with age [6]. Studies have shown that preventing the decline in physical capabilities is crucial for maintaining independent living for older adults [7, 8]. A study reported that locomotion disability is significantly related to poor performance on locomotion tests [9]. Furthermore, decreased muscle strength and ROM reportedly contribute to locomotion-related functional limitations, including performance deterioration [10, 11], which has been observed in older adults with locomotion limitations [9]. Despite the aforementioned findings, to the best of our knowledge, no study has yet examined whether reductions in muscle strength and ROM cause locomotion disability via locomotion-related functional limitation among healthy older adults.

In particular, for older adults, excellent locomotor performance achieved by maintaining muscle strength and lower extremity ROM can potentially prevent locomotion disability and maintain or prolong independent ADLs. Therefore, the present study aimed to determine whether muscle strength and ROM affect locomotion disability via locomotion-related functional limitations in healthy older adults. We hypothesized that reductions in muscle strength and ROM would negatively influence locomotion disability via locomotion-related functional limitations. Confirmation of the applicability of this model to healthy older adults who have yet to report locomotion disability would suggest that interventions focused on maintaining muscle strength and lower extremity ROM should be strongly recommended to prevent locomotion disability.

## 2. Materials and Methods

### 2.1. Study Design

All the measured parameters were essential for assessing the functional status of participants; the measurements were harmless for the participant. All procedures were conducted in accordance with the Declaration of Helsinki, Ethical Principles for Medical Research Involving Human Subjects, adopted by the World Medical Association in 1975. This cross-sectional study was approved by the ethics committee of the Graduate School of Integrated Arts and Science of Hiroshima University (ID: 25-26). All participants provided written informed consent before enrollment.

### 2.2. Participants

This study included older adults residing within Higashi-Hiroshima city and Hiroshima city, Japan. Participants visiting the Higashi-Hiroshima General Welfare Center, Hakuwa Fitness Center, or public community centers were recruited through several channels, including posters, fliers, or visitations to community-based senior citizens' clubs. Recruitment was conducted from December 2011 to October 2012. The inclusion criteria for study participation were as follows: (1) age of 60 years or older, (2) living within the community, (3) capable of performing ADL without any aid, and (4) written informed consent obtained. Individuals diagnosed with severe musculoskeletal, neurological, visual, sensory, or cognitive disorders that may require active management at district hospitals were excluded. Before study initiation, all participants were informed regarding the procedures and their right to freely reject measurements for any reason at any given time.

### 2.3. Sample Size

According to Kline [12], an adequate sample size should be 10 times the number of the parameters in path analysis. Considering that 13 parameters were included in the hypothetical models (described below), at least 130 recruited participants were required.

### 2.4. Outcome Assessment

#### 2.4.1. Anthropometric Measurements

Body height and weight were measured using a standard tape and weighing scale, respectively.

Nagi's model suggests that the pathology for impairment (dysfunctions and structural abnormalities in specific body systems, e.g., physiological functions) leads to functional limitations (e.g., poor performance on locomotion tasks), consequently increasing the risk of disability (expression of a physical limitation) [13]. Based on Nagi's model [13], muscle strength and ROM were measured as indexes of impairment. Moreover, the 10 m hurdle walking test and side-step test were used as indexes of locomotion-related functional limitations, whereas the ADL survey's locomotor domain was used as an index of locomotion disability.

### 2.5. Primary Outcomes

#### 2.5.1. Muscle Strength and ROM as Impairment Indexes

Grip strength was measured twice using a grip meter (TKK5401; Takei Scientific Instruments, Niigata, Japan) in the standing position. The best grip strength results for the right and left hands were recorded.

ROM was measured using the method previously described by Jung and Yamasaki [14]. Hip flexion and knee flexion ROMs were measured as the lower extremity ROM because these ROMs are associated with specific ADLs (e.g., standing up from a sitting position, donning lower body clothing and shoes, or stepping out of a bathtub), suggesting that if there is a reduction in these ROMs, then ADLs will be limited [1, 15]. Prior to the measurements, participants were not allowed to warm up to prevent possible effects on the measurement results. Before ROM measurements, each ROM movement was thoroughly described to the participants. Thereafter, participants moved the joint through the full ROM during which the measurements were obtained by the evaluator. Hip flexion ROM was measured in the supine position, whereas knee flexion ROM was measured in the prone position. When the fixed arm of the goniometer had to be placed perpendicular to the floor, the evaluator visually confirmed the position. The positions within the anatomical landmarks required for ROM measurements were confirmed by the evaluator using hands. If necessary, participants were allowed rest periods between ROM measurements, and the measurements were resumed when the participant indicated that he/she was ready to continue. All ROMs were accurately measured at one time, with mean ROM values for the left and right sides being used for further analysis.

### 2.6. Secondary Outcomes

#### 2.6.1. Locomotor Performance Tests to Assess Locomotion-Related Functional Limitations

For the 10 m hurdle walking test [16], hurdles with a height of 20 cm, width of 10 cm, and length of 100 cm made from Styrofoam were placed at 2 m intervals. As such, a total of six hurdles were placed from the start line (0 m) to the finish line (10 m). The time taken to walk a straight 10 m line from the first step past the hurdle on the start line to the first step past the hurdle on the finish line at normal gait speed was recorded.

For the side-step test [17], a centerline was created and two parallel lines on both sides, 1 m away from the centerline. Participants were instructed to stand at the centerline and then step over the right line with the closest foot, return to the centerline, step over the left line with the closest foot, and then back to the centerline. The number of passes across each line were scored while repeating the above exercise for 20 s.

To familiarize participants with the tests mentioned earlier, several practice trials were provided. The participants were provided sufficient rest periods between trials, and repeat measurements were accordingly obtained. Mean values of the best grip strength results for both hands were calculated. The 10 m hurdle walking test and the side-step test were performed twice, with the best results of both trials being used for further analysis.

#### 2.6.2. Self-Reported Locomotion Disability

The locomotor domain of the ADL survey, which has been used to confirm whether Japanese older adults can safely participate in physical fitness tests administered by the Ministry of Education, Culture, Sports, Science, and Technology of Japan, was selected to assess locomotion disability [18]. Reportedly, the effectiveness of the ADL survey was significantly related to age and physical fitness tests, such as grip strength, sit-and-reach distance, the 10 m hurdle walking, and one-leg standing time with eyes open [19]. Furthermore, self-reported locomotion disabilities were observed in older adults with mild and severe knee pain [18]. The degree of locomotion ability required for independent daily life was evaluated according to the following five items: walking, running, jumping across a ditch, ascending and descending stairs, and carrying a heavy object (Table 1). Each item consisted of three different difficulty levels, with participants selecting the appropriate level for each locomotion ability item. The answers selected by the participants were considered as scores ranging from 1, which was the worst locomotion ability, to 15, which was the best locomotion ability.

### 2.7. Blinding

To control for measurement bias, the first author who designed the study hypothesis did not perform participant recruitment or data collection. The study staff, who collected the data (participant recruitment and measurements), and the participants were not informed of the study hypothesis until completion of the study. RT performed the data analysis.

### 2.8. Statistical Analysis

The normality of data distribution was verified using the Shapiro–Wilk test, whereas the homogeneity of variance was assessed using Levene's test. To compare variables between men and women, independent sample *t*-tests were performed when the data were homogeneous and normally distributed, whereas Mann–Whitney *U* tests were applied whenever normality, and homogeneity of variance were absent. Pearson correlation coefficients were used to calculate correlations among variables. Quantitative variables in this study were included in the following hypothetical model: locomotion disability would be influenced by locomotion-related functional limitations; locomotion-related functional limitations would be influenced by muscle strength and ROM. Accordingly, path analysis was performed to confirm the fitness of the hypothetical model. The goodness of fit of the model was assessed using statistical parameters, such as the chi-square value, goodness of fit index (GFI), adjusted goodness of fit index (AGFI), comparative fit index (CFI), and root mean square error of approximation (RMSEA). A low chi-square value relative to the degrees of freedom with an insignificant *p* value (*p* > 0.05), GFI value of >0.90, AGFI value of >0.90, CFI value of >0.95, or RMSEA value of <0.06 indicated good model fit [20]. Statistical analyses were performed using IBM SPSS statistics 22 and IBM SPSS Missing Values (IBM SPSS, Tokyo, Japan) and AMOS 16.0.1 [21].

## 3. Results

**Figure 1:**
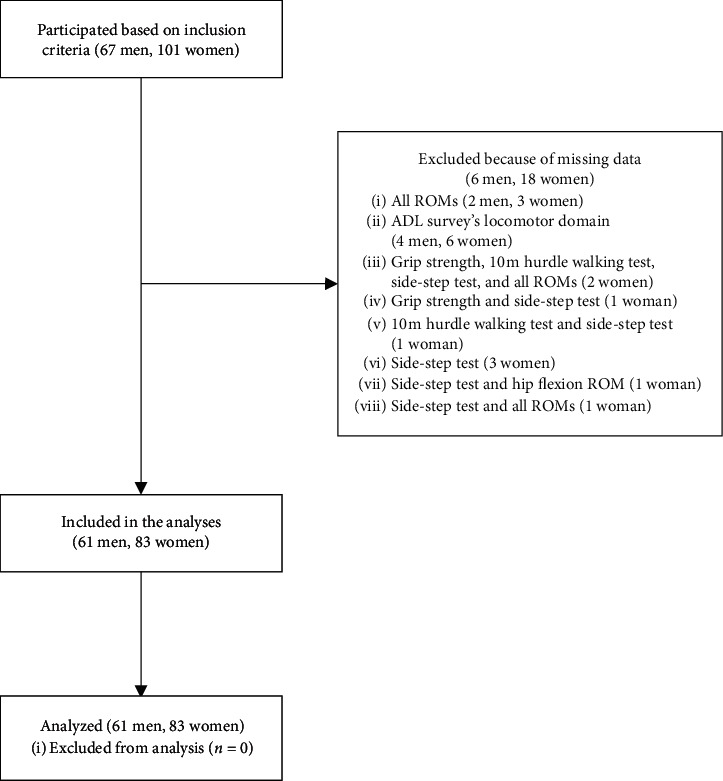
Flow diagram of the selection process of the participants. ADL, activities of daily living. ROM, range of motion.

Overall, 168 older adults volunteered for the present study based on the inclusion criteria. However, 24 individuals were excluded owing to missing data. Thus, a total of 144 participants (61 men, 83 women) were analyzed (Figure 1).

Table 2 presents the result of the comparison between older men and women in terms of physical characteristics, grip strength, ROM, locomotor performance test scores, and the ADL survey's locomotor domain total scores. Accordingly, compared to older women, older men exhibited significantly higher grip strength and the ADL survey's locomotor domain total scores (*p* < 0.001) as well as a shorter 10 m hurdle walking test time (*p* < 0.001). However, knee flexion ROM in older men was narrower than that in older women (*p*=0.03).

Table 3 presents the pairwise Pearson correlations among grip strength, ROMs, locomotor performance test scores, and the ADL survey's locomotor domain total scores in older men. Hip flexion ROM and the 10 m hurdle walking test time were significantly correlated with each other (*r* = −0.365; *p*=0.004). Correlations were observed between the ADL survey's locomotor domain total scores and grip strength, the 10 m hurdle walking test time, and the side-step test scores (*r* = −0.391 to 0.490; *p* < 0.01).

Pearson correlations for grip strength, ROMs, locomotor performance test scores, and the ADL survey's locomotor domain total scores in older women are presented in Table 4. Hip flexion and knee flexion ROMs were correlated with the 10 m hurdle walking test time (*r* = −0.299 to −0.257; *p* < 0.05). In addition, knee flexion ROM was correlated with the side-step test scores (*r* = 0.292; *p*=0.007). Significant correlations were noted between the ADL survey's locomotor domain total scores and both the 10 m hurdle walking test time and the side-step test scores (*r* = −0.473 to 0.399; *p* < 0.001).

**Figure 2:**
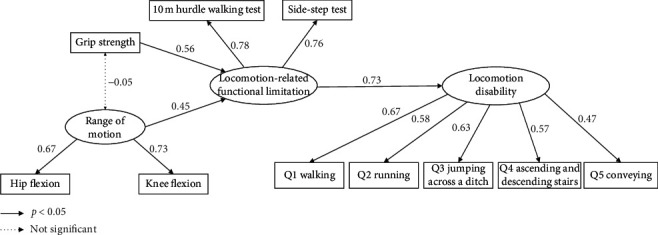
Result of path analysis for the hypothetical model.

Path analysis results for the hypothetical model are presented in Figure 2. In the present study, path analysis was conducted by treating our samples as a general population of older adults, considering the decreased sample size and increased beta error when separating men and women samples. The chi-square value was not significant (chi-square = 41.885, *p*=0.113), whereas the indices of goodness of fit were high (GFI = 0.944, AGFI = 0.904; CFI = 0.970, RMSEA = 0.046), indicating the validity of the model. The result of this analysis showed that locomotion disability was significantly influenced by locomotion-related functional limitations, which, in turn, were influenced by grip strength and lower extremity ROM. Overall, the obtained result is concordant with the hypothesis that locomotion disability was influenced by muscle strength and ROM via locomotion-related functional limitations.

## 4. Discussion

The present study focused on physical capabilities, such as muscle strength and ROM, locomotor performance, and locomotion disability. Our hypothesized model suggested that reductions in muscle strength and ROM negatively influence locomotion disability via locomotion-related functional limitations. Overall, our path analysis supported this hypothesis.

Locomotion limitations have been associated with reduced social participation, disability, nursing home institutionalization, and death [22–24]. Lower locomotor performances have been observed in older adults who self-reported at least some locomotion difficulty/disability, such as walking one-quarter of a mile and climbing 10 steps [9]. However, no study has yet examined the relationships among muscle strength and ROM, locomotion-related functional limitation, and locomotion disability in healthy older adults. Our study showed that locomotion-related functional limitation influenced locomotion disability. Moreover, muscle strength and ROM influenced locomotion disability via locomotion-related functional limitation. All but 9 participants in our study were able to safely perform the side-step test, which examines the ability to quickly step sideways—an action that several older adults find difficult to execute [25, 26]. Therefore, the participants can be considered very healthy. Among healthy older adults without ADL disability, validation of the disability construct developed in our study, including impairment, functional limitation, and disability defined in Nagi's model, is a novel finding that contributes to geriatric research. Considering that older adults with ADL disabilities have difficulty restoring their ability to perform ADLs independently [27], our results indicate that exercise interventions, even for healthy older adults with no functional limitations, should focus on maintaining muscle strength and lower extremity ROM.

The present study highlights the significant relationships between muscle strength, ROM, and locomotion ability. Decreased muscle strength and ROM limitations are considered predictors for assisted living or skilled nursing facility admission among older adults [28]. A 2-year follow-up study of older adults who had reported the absence of ADL difficulties/disabilities at baseline found that the onset of difficulty/disability in waking was the highest (14.3%) among all ADLs and was followed by other ADLs, such as bathing, dressing, eating, getting in/out of chairs, or using the toilet [29]. Furthermore, a cross-sectional study reported that restricted hip and knee flexion ROMs were associated with slow walking speeds in a sample of community-dwelling older adults [10]. Slow walking speeds have been correlated with weak grip strength [30]. Therefore, our results as well as those of previous studies suggest that decreased muscle strength and ROM can affect locomotion limitations in older adults.

Our results have potentially important implications for older adults. Considering that muscle strength and ROM improvements can increase locomotion ability in older adults, exercise interventions may play a crucial role in increasing muscle strength and ROM, thereby improving the locomotion ability required for functional independence [31]. Furthermore, associations between locomotion limitations and disability, loss of independence, and mortality have been reported in the literature [30, 31]. Muscle strength and ROM exercises that improve locomotion ability are warranted, considering that improvements therein may confer broader benefits to the well-being of older adults. Based on the findings of the present study, older adults should be encouraged to participate in physical exercise programs that focus on strengthening muscles and improving ROM to counteract age-related locomotion disability/difficulty.

Our study has several limitations worth mentioning. First, this study did not include all muscle strength and lower extremity ROM measurements as variables. However, grip strength was considered an index representative of overall muscle strength [32]. Furthermore, a previous study reported that hip and knee flexion ROMs significantly contribute to walking velocity in older adults [10]. Therefore, the significant association between muscle strength and ROM and locomotion ability obtained in our study can be applicable to older adults. Second, this study cannot determine a strict causal relationship owing to the cross-sectional design. Third, our study could not conduct path analysis separately for men and women because of the small sample size. Further studies including a large sample sizes would be required to establish a comparison between men and women.

## 5. Conclusions

The current study demonstrated that locomotion disability was influenced by muscle strength and ROM via locomotion-related functional limitations. Further longitudinal studies among healthy older adults are required to examine the manner in which exercise-induced increases in muscle strength and ROM could help maintain or prolong locomotion ability during ADL through increased locomotor performance.

## Figures and Tables

**Table 1:** ADL survey's locomotor domain.

Locomotor domain items	Locomotor domain items answered number^a^
Q1 walking—how long can you walk without taking a rest?	(1) about 5–10 minutes
	(2) about 20–40 minutes
	(3) over an hour

Q2 running—how long can you run without taking a rest?	(1) impossible
	(2) about 3–5 minutes
	(3) over 10 minutes

Q3 jumping across a ditch—how wide a gutter can you jump over?	(1) impossible
	(2) about 30 cm
	(3) about 50 cm

Q4 ascending and descending stairs—how do you go up the stairs?	(1) need banister
	(2) slowly (no use banister)
	(3) fleetly (no use banister)

Q5 carrying an object—what weight of baggage can you carry 10 m?	(1) impossible
	(2) about 5 kg
	(3) about 10 kg

ADL, activities of daily living; ^a^the numbers answered are scores.

**Table 2:** Physical characteristics, grip strength, range of motion, locomotor performance test scores, and ADL survey's locomotor domain (mean ± SD).

Variables	Entire sample (*n* = 144)	Men (*n* = 61)	Women (*n* = 83)	*p* value
Age (years)	70.6 ± 5.9	71.1 ± 6.0	70.1 ± 5.9	0.312^a^
Height (cm)	157.2 ± 8.2	164.1 ± 6.3	152.2 ± 5.3	<0.001
Weight (kg)	56.3 ± 9.2	61.5 ± 8.0	52.5 ± 8.0	<0.001
BMI (kg/m^2^)	22.7 ± 2.9	22.8 ± 2.3	22.6 ± 3.2	0.328^a^

Grip strength (kg)	30.3 ± 9.3	39.2 ± 6.9	23.8 ± 3.6	<0.001^a^
*Range of motion*				
Hip flexion (degrees)	118.2 ± 9.7	117.1 ± 8.3	119.0 ± 10.6	0.247
Knee flexion (degrees)	24.8 ± 8.2	123.6 ± 8.1	126.0 ± 8.6	0.030^a^
*Locomotor performance test scores*				
10 m hurdle walking test (seconds)	6.7 ± 1.6	6.4 ± 2.0	6.9 ± 1.3	<0.001^a^
Side-step test (scores)	32.4 ± 6.8	33.7 ± 7.2	31.5 ± 6.3	0.055

ADL survey's locomotor domain total scores	12.7 ± 2.0	13.4 ± 1.6	12.2 ± 2.1	<0.001^a^
Q1 (scores)	2.6 ± 0.6	2.7 ± 0.5	2.5 ± 0.6	0.040^a^
Q2 (scores)	2.2 ± 0.7	2.3 ± 0.7	2.1 ± 0.7	0.086^a^
Q3 (scores)	2.6 ± 0.6	2.8 ± 0.4	2.5 ± 0.6	0.003^a^
Q4 (scores)	2.6 ± 0.5	2.7 ± 0.5	2.6 ± 0.6	0.342^a^
Q5 (scores)	2.7 ± 0.5	2.9 ± 0.3	2.5 ± 0.5	<0.001^a^

ADL, activities of daily living; ^a^the Mann–Whitney *U* test.

**Table 3:** Pearson correlations between variables in older men (*n* = 61).

Variables	Hip flexion range of motion		Knee flexion range of motion		10 m hurdle walking test		Side-step test		ADL survey's locomotor domain total scores	
	*r*	*p* value	*r*	*p* value	*r*	*p* value	*r*	*p* value	*r*	*p* value
Grip strength	0.413	<0.001	0.145	0.265	−0.550	<0.001	0.589	<0.001	0.333	0.009
Hip flexion range of motion			0.439	<0.001	−0.365	0.004	0.188	0.148	0.149	0.250
Knee flexion range of motion					−0.215	0.097	0.173	0.181	0.035	0.787
10 m hurdle walking test							−0.668	<0.001	−0.391	0.002
Side-step test									0.490	<0.001

ADL, activities of daily living.

**Table 4:** Pearson correlations between variables in older women (*n* = 83).

Variables	Hip flexion range of motion		Knee flexion range of motion		10 m hurdle walking test		Side-step test		ADL survey's locomotor domain total scores	
	*r*	*p* value	*r*	*p* value	*r*	*p* value	*r*	*p* value	*r*	*p* value
Grip strength	0.026	0.819	−0.077	0.489	−0.342	0.002	0.320	0.003	0.278	0.011
Hip flexion range of motion			0.516	<0.001	−0.257	0.019	0.230	0.037	0.391	<0.001
Knee flexion range of motion					−0.299	0.006	0.292	0.007	0.289	0.008
10 m hurdle walking test							−0.531	<0.001	−0.473	<0.001
Side-step test									0.399	<0.001

ADL, activities of daily living.

## Data Availability

The data used to support the findings of this study are available from the corresponding author upon reasonable request.
